# Determination of protein transporter function using Raman spectroscopy

**DOI:** 10.1099/mic.0.001526

**Published:** 2025-02-10

**Authors:** Dominic Gilchrist, Meez Islam, Muhammad Safwan Akram, Paul Dean

**Affiliations:** 1 School of Health and Life Sciences, Teesside University, Campus Heart, Middlesbrough TS1 3BX, UK; 2 National Horizons Centre, 38 John Dixon Lane, Darlington DL1 1HG, UK

**Keywords:** alkyne, ATP, *Microsporidia*, Nucleotide transporter, Raman spectroscopy, uptake assay

## Abstract

Transporter proteins are essential across the tree of life as they provide a cell with a means of exchanging vital metabolites with the external milieu. Characterizing the function of transporters is challenging and traditionally uses methods involving radiolabelled substrates, which requires prolonged exposure times and specialist equipment. Here, we provide an alternative method to the classical uptake assay using Raman spectroscopy to detect the uptake of alkyne-labelled substrates and determine transporter function. As a proof of principle, we demonstrate the method using a candidate nucleotide transporter (ThNTT4) expressed in *Escherichia coli*, which is shown to transport alkyne-labelled ATP molecules (N6pATP), which was readily detected using Raman spectroscopy. We show that ATP transport can be detected in a time-dependent manner using alkyne labels and demonstrate the substrate specificity of the transporter for purine but not pyrimidine substrates. This work establishes that Raman spectroscopy is an excellent alternative to using radioactive substrates in analysing, not only pathogen transporters, but potentially any transporter in which its substrate can be alkyne tagged.

## Graphical Abstract

A summary of the workflow for identifying the function of a protein transporter using Raman spectroscopy. A putative transporter gene (ThNTT4) is cloned into a suitable expression vector [1] and transformed into a heterologous expression system [2]. Protein expression is induced [3] and confirmed [4], followed by an uptake assay using alkyne-labelled substrates such as N6pATP [5]. Following washing and lysis of the heterologous host cell [6], the lysate is spotted onto a steel slide, dried down and analysed with the Raman microscope.

**Impact statement**

Protein transporters are key to the processes of life but are difficult to investigate and determine functionality. This has traditionally involved ‘uptake assays’ using radiolabelled substrates to determine substrate specificity. Here, we report an alternative method to determine substrate uptake that involves the detection of alkyne-labelled substrates and Raman spectroscopy. Our analysis utilizes the heterologous expression of an nucleotide transporter (ThNTT4) as a proof of principle and shows that the transport of alkyne-labelled ATP can be readily detected. This method of substrate detection offers a safer and more accessible alternative to the classical uptake assay and can be applied to potentially any transporter to which a substrate can be alkyne tagged.

## Introduction

Membrane transporter proteins mediate the transport of a range of substrates into a cell (including sugars, proteins, ions, nucleotides and various drugs). They are vital to all cells and represent up to 30% of the human genome with their malfunction linked to many diseases [1,2]. They are also essential virulence factors of medically important human pathogens [3] as they permit the delivery of virulence factors into host cells [4] or the stealing of host-derived nutrients such as nucleotides [5,6]. Because of their importance and abundance, transporter proteins are a logical choice for drug targets, and therefore, understanding their biological function is important.

Determining the function of transporter proteins is inherently difficult and involves sensitive detection methods. The classical ‘uptake assay’ is used to identify the substrate of transporters and involves expressing the transporter gene in a heterologous host such as mammalian cell lines, yeast, *Escherichia coli* or reconstituted liposomes [7,10]. Such tractable expression systems are needed because of the naturally low levels of endogenous transporters in native cell types [9,11], and therefore, over-expression ensures transporter function can be detected. The classical uptake assay involves analysing the uptake of radiolabelled substrates in a heterologous host to ensure high sensitivity of detection. However, the use of radiation in traditional uptake methods comes at a cost due to accessibility and safety issues including the need for a dedicated radiation room with specialized equipment, training, inability to radiolabel particular substrates and prolonged exposure to radiation due to the considerable number of repeated measurements required in uptake assays [5].

Raman spectroscopy provides a means for detecting a wide range of molecules using their light-scattering properties. Each molecule has an identifiable fingerprint, or spectrum, according to the chemical bonds within it [12]. Raman spectroscopy of an entire bacterial cell produces a complex Raman spectrum, which is inherently difficult to decipher and identify specific metabolites [13]. To address these issues, small non-biological tags that are not normally present in nature, such as the triple-bonded carbon alkynes, can be used [14]. The alkyne triple bond exhibits a large Raman peak within the biologically ‘silent region’ of the Raman spectrum (1800–2700 cm^−1^), making alkyne-tagged molecules clearly identifiable and unobscured by other biological molecules [14]. Alkynes are extremely small tags, possessing only two atoms of carbon, and as they are non-functional within a cell and non-reactive with native biomolecules, they provide an excellent tool. Therefore, an alkyne-tagged substrate may provide a novel way to interrogate protein transporters using Raman spectroscopy.

Here, we use a previously characterized nucleotide transporter (NTT) protein from a microsporidian parasite, which has been shown to transport the ATP and GTP purine nucleotides. Using Raman spectroscopy, we show that alkyne-labelled ATP substrates can be readily detected within the *E. coli* cell following the expression of the NTT transporter gene, whereas labelled pyrimidine substrates are not, highlighting the specificity of the detection method. The results show that potentially, any expressed transporter gene could be investigated where the transported substrate can be alkyne tagged.

## Methods

### Bacterial strains and plasmids

The bacterial strain used in this study was *E. coli* BL21(DE3) pLysS (Promega), and the plasmid was pET16b. Transporter genes (ThNTT1-4), cloned into pET16b as His-tagged variants, were a gift from Professor Robert Hirt (Newcastle University), constructed as described previously [15].

### NTT expression and Western blotting

Induction of NTT expression was similar to that described by Dean *et al*. [5]. The NTT genes were expressed from a pET16b vector in freshly transformed *E. coli* BL21(DE3) pLysS. Bacteria were cultured in lysogeny broth (LB) in the presence of chloramphenicol (34 µg ml^−1^) and carbenicillin (100 µg ml^−1^). Single colonies were used to inoculate LB and were incubated at 37 °C with 200 r.p.m. overnight, before inoculation into terrific broth at 0.1 OD_600_. Upon reaching 0.4–0.6 OD_600_, bacteria were rapidly chilled to 18 °C followed by the addition of 1 mM IPTG to induce NTT gene expression. Expressing strains were incubated for an additional 16–18 h at 18 °C with 200 r.p.m. to permit adequate NTT expression. Bacteria were then kept chilled on ice and pelleted at 14 000 ***g*** for 1 min, before being lysed in 2% SDS plus 1× nuclease/protease inhibitor cocktail mix. The lysate was then centrifuged at 14 000 ***g*** for 5 min, and the protein concentration of the supernatant was determined by BCA reagent (ThermoFisher). Twenty micrograms of total protein of the supernatant were resolved by SDS-PAGE, which were blotted onto PVDF membrane and were detected with an anti-His primary antibody (Sigma) using colorimetric analysis and densitometry. The expression of the NTTs was routinely confirmed during the uptake assays, and only expressing strains were used in the uptake assays.

### Transporter assay using Raman spectroscopy

Alkyne-labelled ATP [N6pATP or 2-ethynyl-ATP (2-EATP), Sigma] and the alkyne-labelled thymidine analogue 5-Ethynyl-2′-deoxyuridine (5-EdU, Jena Bioscience) were used in uptake assays. 5-Edu is a labelled thymidine mimic that readily incorporates into DNA and has been well characterized previously [16]. Based on expression data (Fig. S1, available in the online Supplementary Material), we used the gene ThNTT4 as the prototypical gene for the uptake assay. Bacteria expressing ThNTT4 were washed twice in PBS at pH 6.8 and adjusted to an OD_600_ of 5.0 and thereafter kept at 4 °C prior to experiments. *E. coli* BL21(DE3) pLysS cells containing an empty pET16b vector were used as a negative control. Uptake was performed using parameters as described previously [6]. One hundred microlitres of PBS containing 0.5 µM non-labelled ATP (or thymidine analogue EdU) were spiked with 1 nM alkyne-labelled substrates. Bacteria and uptake media were pre-warmed to 25 °C for 10 min after which 100 µl of bacteria was added to 100 µl of the uptake medium and incubated at 25 °C with shaking at 200 r.p.m. After 10 min, the substrate transport was complete and the reaction was quenched with 1 ml of ice-cold PBS containing 250 µM of the relevant non-labelled substrate. Bacteria were quickly washed using PBS and non-labelled substrate, and the resulting pellet was lysed in 50 µl lysis buffer (2% SDS, nucleases and protease inhibitors) for 1 h. The total lysate was spotted on a stainless steel slide (Renishaw) and air dried prior to Raman microscopy analysis. Competition assays between substrates were carried out using a 1-million-fold excess of non-labelled substrate compared to alkyne-labelled ATP as described previously [5]. Uptake levels were assessed by analysing the area under the respective peaks and compared using a t-test for unequal variance.

### Raman microscopy

All Raman microscopy measurements were carried out using a Renishaw inVia Qontor confocal Raman microscope with a 532 mW laser. Spectra were recorded at 1 cm^−1^ resolution using a 2400 lines/mm grating and captured with a 50× objective lens. Samples were prepared by dispensing 50 µl lysate onto a stainless steel slide and air dried before Raman detection. All samples were measured from 20 random points, and from each point, a 5-s integration time and 20 accumulations were taken. Measurements were taken in two parts, between 600 and 1700 cm^−1^, to confirm the presence of biological spectra, followed by scans of 2000 to 2300 cm^−1^ within the biological silent region for detection of the alkyne peak. The detectable peak between 2179 and 2185 cm^−1^ was determined experimentally to correspond to the N6pATP alkyne peak. Post-spectral analysis was completed using WIRE software (Renishaw). Cosmic ray and background signal removal was performed prior to curve fitting, which was carried out using the software.

## Results and discussion

### Raman spectroscopy detection of alkyne-labelled substrates

**Fig. 1.**
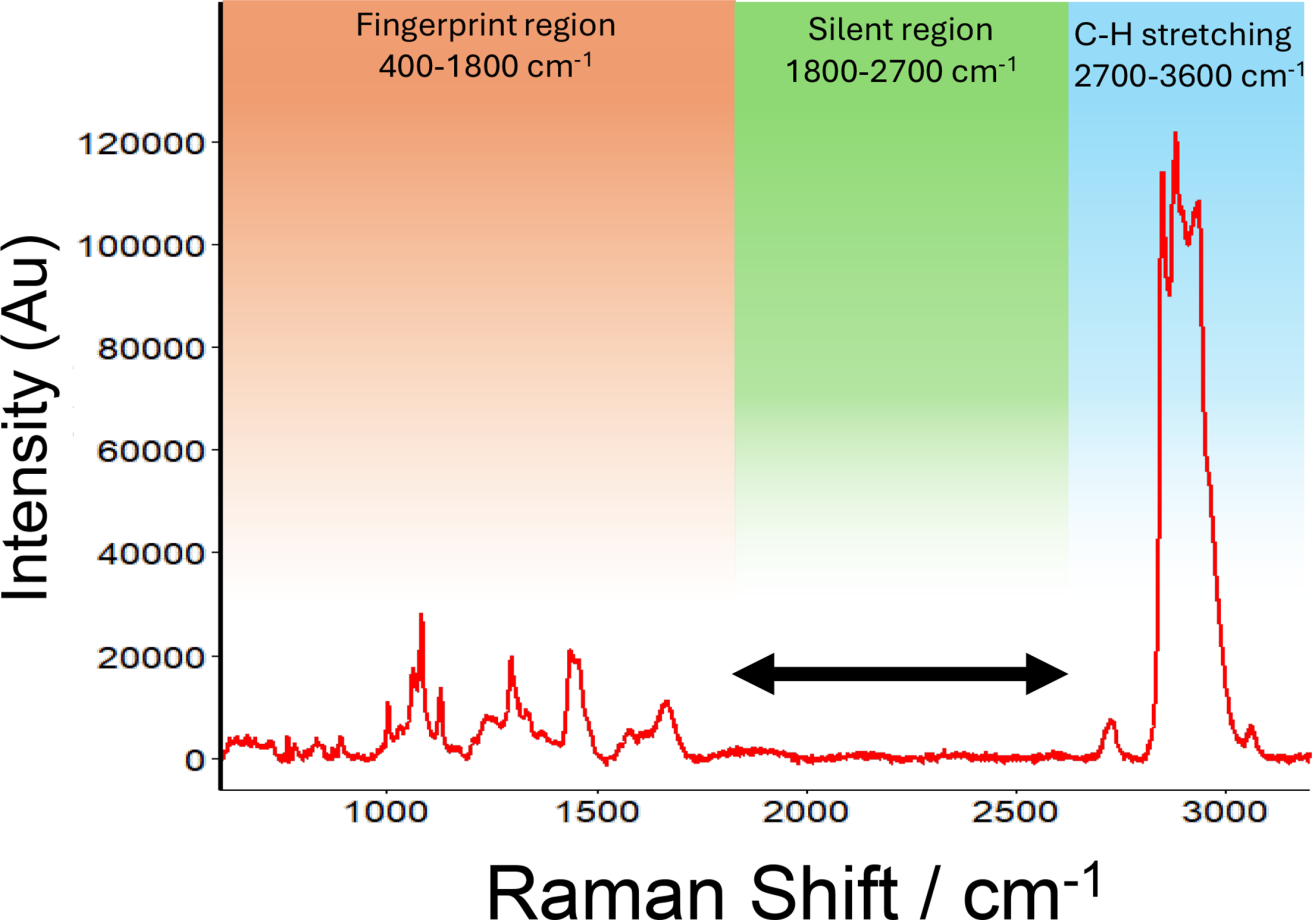
Representative Raman spectrum of the *E. coli* BL21 pLyS cell lysate. The lysate was air dried and spotted onto mirrored stainless steel slides and analysed as described in the Methods section. The green range in the image highlights the biological silent region within which there are few peaks recorded.

**Fig. 2.**
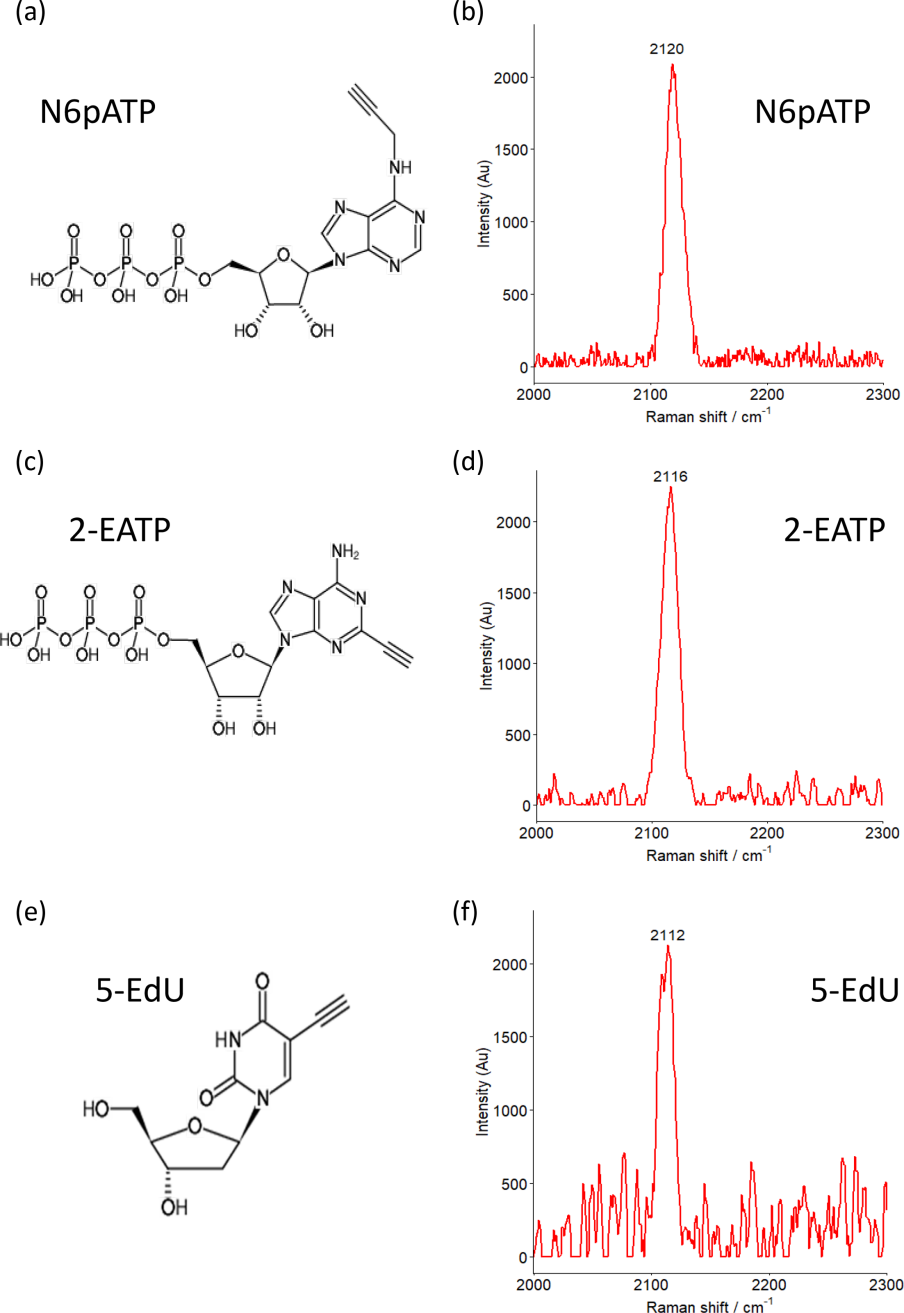
Analysis of alkyne-labelled substrates The alkyne-labelled substrates utilized in this study are shown (a, c and e). Raman spectra were collected from 2 µl of 2 mM standards air dried onto mirrored stainless steel slides. N6pATP (a) produced a distinct peak at 2120 cm^−1^ (b), 2-EATP (c) produced a distinct peak at 2115 cm^−1^ (d) and the thymidine analogue 5-EdU (e) gave a peak at 2112 cm^−1^ (f). All measurements were captured using a Raman confocal microscope with a 532 nm laser at 50% power and with a 50× objective with a 5-s acquisition time and 10 accumulations. Post-sampling analysis included cosmic ray and background removal using Renishaw WIRE software. AU, arbitrary unit.

Due to the complexity of a bacterial cell, the biological region of the Raman spectrum is inherently complicated, and the detection of specific substrates is difficult in this region, as shown with the lysate of *E. coli* BL21 (Fig. 1). Alkyne labels can be detected within the biological silent region [14] between 1800 and 2700 cm^−1^ (Fig. 1), and therefore, alkyne-labelled ATP could be readily detected in an *E. coli* lysate (Fig. 1). Two alkyne-labelled ATP molecules were tested in this study along with one alkyne-labelled thymidine analogue (Edu), which were readily detected within the Raman spectrum (Fig. 2). The alkyne-labelled ATP analogue N6-propargyl-ATP (N6pATP) (Fig. 2a) produced a Raman peak at ~2120 cm^−1^ (Fig. 2b), and to assess the effect of alkyne positioning on ATP, 2-EATP (Fig. 2c) was also utilized, which produced a peak at 2115 cm^−1^ (Fig. 2d). As a negative control in the uptake assay, we utilized the thymidine analogue 5-EdU (a pyrimidine not reported to be transported by ThNTT4 [5] (Fig. 2e), which produced a peak at 2112 cm^−1^ (Fig. 2f). All three alkyne-labelled substrates produced clear peaks within the biological silent region, making them readily distinguishable, in line with results from previous studies of alkyne tags [14] and therefore suitable substrates for the transporter assays.

### Identifying a reliable NTT transporter for uptake assay testing

To identify a candidate transporter for use in the uptake assay, we tested four NTT proteins (ThNTT1-4) from the microsporidian parasite *Trachipleistophora hominis* that have been previously shown to transport ATP [5,15]. Gene expression and protein stability were assessed by Western blot, which showed that ThNTT4 was consistently expressed in *E. coli* BL21, above and beyond the other transporters (ThNTT1,2,3), and therefore, this protein was chosen as the candidate transporter for the uptake assay (Fig. S1). ThNTT3 expression was consistently low, in line with the previous findings [5].

### Transport of alkyne-labelled substrates in *E. coli* expressing ThNTT4

**Fig. 3.**
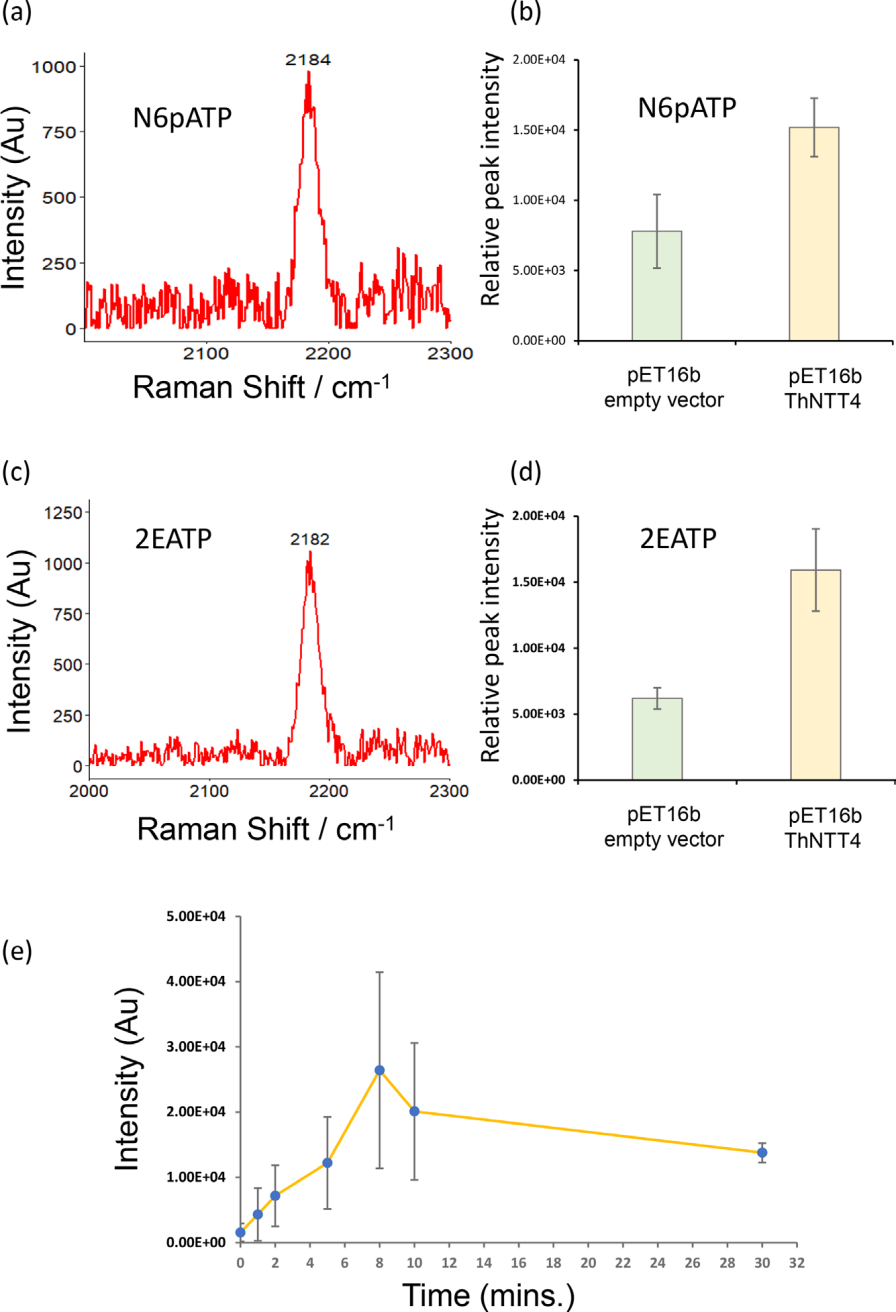
Uptake of alkyne-labelled substrates by *E. coli* expressing ThNTT4. Bacteria were prepared for uptake as described in the Methods section. Transport of N6pATP was determined following bacterial lysis and evaluation by Raman spectroscopy. (a) Representative Raman peak of N6pATP following uptake by *E. coli* expressing ThNTT4 detected a shift in the Raman shift to 2184 cm^−1^. (b) N6pATP uptake by *E. coli* expressing ThNTT4 was significantly higher than that of the empty vector control (*P*<0.05). (c) Representative Raman peak of 2-EATP following uptake by *E. coli* detected at a Raman shift of 2182 cm^−1^. (d) Uptake of 2-EATP by *E. coli* expressing ThNTT4 exhibited a significantly higher uptake than that of the empty vector control (*P*<0.05). (e) Time course experiment N6pATP uptake by *E. coli* expressing ThNTT4 taken between 0 and 30 min. All samples were air dried onto mirrored stainless steel slides, and 20 measurements were taken from random points. For each measurement, 5s exposure and 10 accumulations were taken. The data were processed using WIRE software. Data show mean ± sd. The uptake of N6pATP was across five biological replicates; all further experiments account for three biological replicates. Raman intensity values represent the area under the curve for each Raman peak. AU, arbitrary unit.

Traditional uptake assays using radiolabelled substrates have previously identified that ThNTT4 transports the purine nucleotide ATP, but not pyrimidine nucleotide or nucleosides such as UTP, TTP or thymidine [5,15]. Uptake assays with alkyne-labelled substrates were performed using culture conditions similar to those previously described with radiolabelled substrates [5,6, 15]. The ThNTT4 transporter was expressed in *E. coli*, and uptake assays were performed with alkyne-labelled ATP (N6pATP). The data show a clear definable peak corresponding to the N6pATP signal in the *E. coli* lysate, significantly above background levels following ThNTT4 expression compared to the empty vector (Fig. 3a, *P*<0.05). The slight shift in the expected peak in the silent region from 2120 to 2184 cm^−1^ (Fig. 3a) is a phenomenon that has been reported previously due to the transfer to a more hydrophobic intracellular environment and incorporation into macromolecules [17]. Similar ATP uptakes were seen with the labelled alkyne variant 2-EATP (Fig. 3d, *P*<0.05), which showed a similar shift from the initial peak produced at 2115 to 2182 cm^−1^ (Fig. 3c). A time course experiment using the Raman uptake method showed that the transport of alkyne-labelled ATP increased over time, reaching the highest recorded uptake at the 8-min time point, similar to that previously shown for ThNTT4 (Fig. 3e) in a previous study [15]. These data clearly show that Raman spectroscopy can detect alkyne-labelled ATP substrates transported by the NTT protein.

### Determining specificity of the ThNTT4 transporter using Raman microscopy

**Fig. 4.**
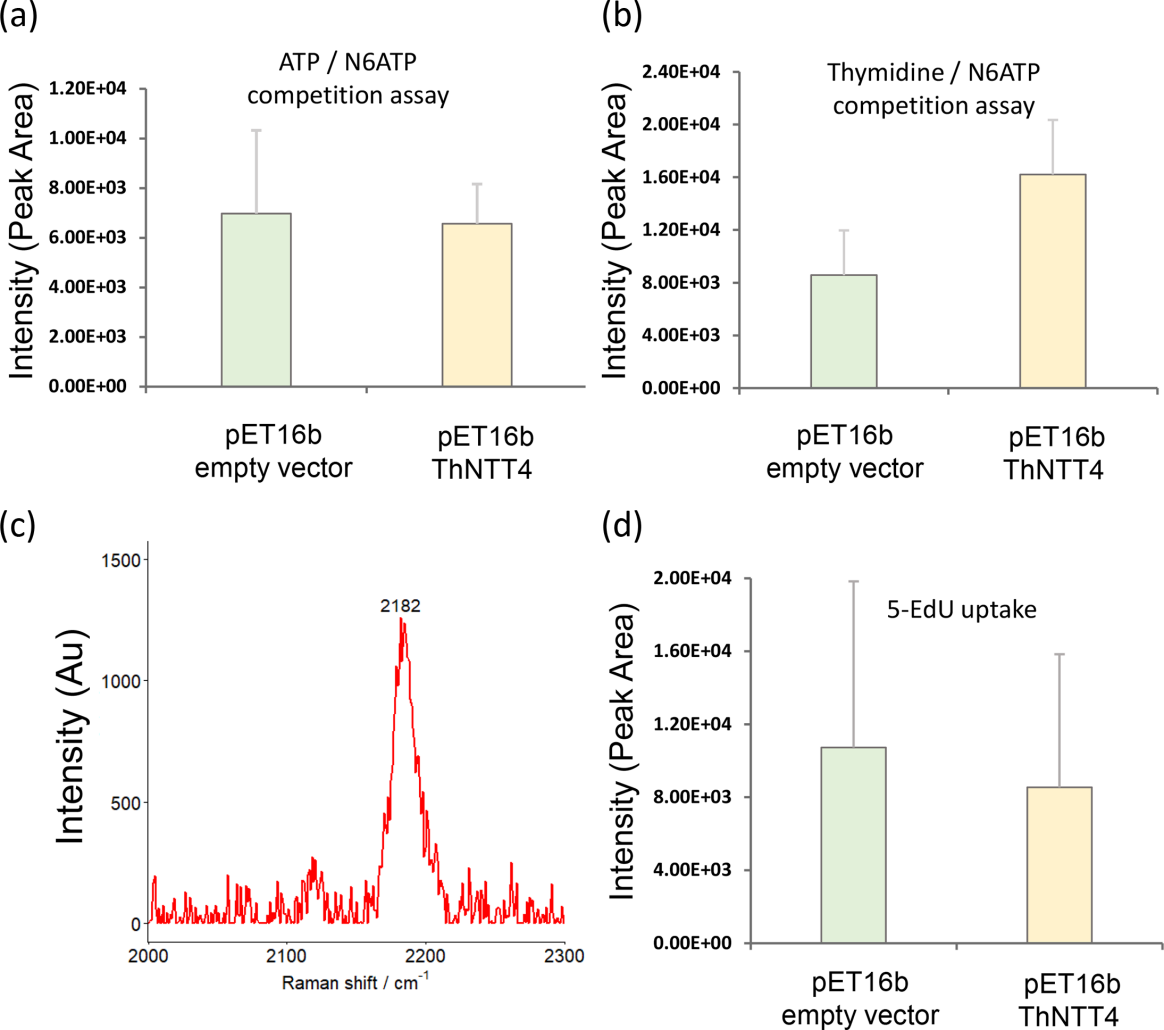
Competition assay and 5-Edu uptake in *E. coli* expressing ThNTT4. (**a**) Uptake of N6pATP by *E. coli* expressing ThNTT4 in the presence of a 1-million-fold excess of extracellular unlabelled ATP. No uptake of N6ATP was observed compared to that of the empty pET-16b vector control (*P*>0.05). (**b**) Uptake of N6pATP by *E. coli* expressing ThNTT4 in the presence of a 1-million-fold excess of extracellular unlabelled pyrimidine thymidine showed a significant increase in uptake compared to the empty pET-16b control (*P*<0.05). (**c**) Representative Raman peak of 5-Edu in an *E. coli* lysate which detected a Raman shift of 2182 cm^−1^. (**d**) Raman uptake of 5-Edu by *E. coli* expressing ThNTT4 revealed no significant increase compared to the empty vector control (*P*>0.05). All samples were air dried onto mirrored stainless steel slides, and 20 measurements were taken from different points around the dried lysate. For each measurement, 5s exposure and 10 accumulations were taken. Data show mean ± sd. The uptake of N6pATP was across five biological replicates; all further experiments account for three biological replicates. Raman intensity values represent the area under the curve for each Raman peak. AU, arbitrary unit.

Competition assays are an important measure of robustness in transporter studies, and as ThNTT4 has been shown unable to transport pyrimidine nucleotides or nucleosides [5,6, 15], this offered an opportunity to test the specificity of the uptake method. A competition assay was performed using an excess of unlabelled ATP to saturate the transport of the alkyne-labelled N6pATP, similar to that performed previously with radiolabelled substrates [5]. The incubation of the bacteria in the presence of 1-million-fold excess of unlabelled ATP reduced the transport N6pATP to background levels, with no significant difference between ThNTT4 and the empty pET16b vector control (Fig. 4a, *P*>0.05). When non-labelled ATP was replaced with non-labelled thymidine (that is not transported by ThNTT4), the transport was not significantly inhibited (*P*<0.05), showing specificity for ATP transport and highlighting the robustness of the assay (Fig. 4b)

Finally, the substrate specificity of the ThNTT4 transporter was further supported as ThNTT4 was unable to transport the alkyne-labelled pyrimidine nucleoside analogue 5-EdU in line with the previous work using radiolabelled substrates [5]. There was no significant uptake of 5-EdU observed by ThNTT4 above that of the control (Fig. 4d, *P*<0.05), unlike that of ATP (Fig. 3a). Taken together, these data demonstrate that Raman spectroscopy in conjunction with alkyne-labelled substrates provides a reliable alternative to the use of radiolabelled substrates in interrogating protein transporters.

## Conclusions

Progress in understanding transporter function has been hampered by methodological barriers including the low levels of native expression and the need for highly sensitive detection methods involving radioisotopes. Radio-labelled substrates have been used for decades in traditional transporter ‘uptake assays’ to determine transporter function due to their high sensitivity of detection; however, the risks from prolonged use and the additional costs of disposal and dedicated facilities pose a barrier to their use and in turn hinder the advancement in our knowledge of transporter proteins.

Here, we have shown that Raman spectroscopy offers a new avenue to interrogate transporter proteins without the need for radioisotopes. Our candidate transporter, ThNTT4, was shown to consistently transport alkyne-labelled ATP in a time-dependent manner. In comparison to the traditional uptake assay, the volumes and amounts of cells required are identical, so is the amount of labelled ATP [5]. The time taken to perform the Raman uptake assay is also comparable to the traditional method albeit slightly longer. However, with optimization and refinement of the method, the time taken could be reduced.

The described method, which we illustrate in Fig. S2, could be applied to a wide range of transporters because alkyne-labelled substrates are becoming more readily available, for example, due to their popular use in click chemistry. Importantly, as alkynes are extremely rare in nature [18] and only detected in the biological silent region of the Raman spectrum, they can be readily detected above the levels of other biomolecules – making them excellent labels for transporter substrates. They have a very small size and have minimal impact on the parent molecule. Fluorescent labelling is not a viable alternative as this is greatly hindered by the size of the fluorescent molecule, which can impact the biological function of the substrate [14].

Further modifications of the proposed method are possible such as the use of nanoparticles (gold or silver) that could be employed to enhance the Raman signal, further increasing the sensitivity [19]. While the method presented here focussed on the parasite transporter ThNTT4 in an *E. coli* heterologous cell, this method is potentially applicable to several cell systems such as yeast*, Lactococcus*, *Xenopus* and even liposomes, for which radiolabelled substrates have been used.

## Supplementary material

10.1099/mic.0.001526 Uncited Supplementary Material 1.

## Abbreviations

2-EATP: 2-ethynyl-ATP
5-EdU: 5-ethynyl-2′-deoxyuridine
LB: lysogeny broth
NTT: nucleotide transporter
