# Supplementary material for: Determination of protein transporter function using Raman spectroscopy
Source: Microbiology (Reading). 2025 Feb 10;171(2):001526. doi: 10.1099/mic.0.001526 (PMC12282265; doi:10.1099/mic.0.001526)
Supplement: Uncited Supplementary Material 1. [file mic-171-01526-s001.pdf]

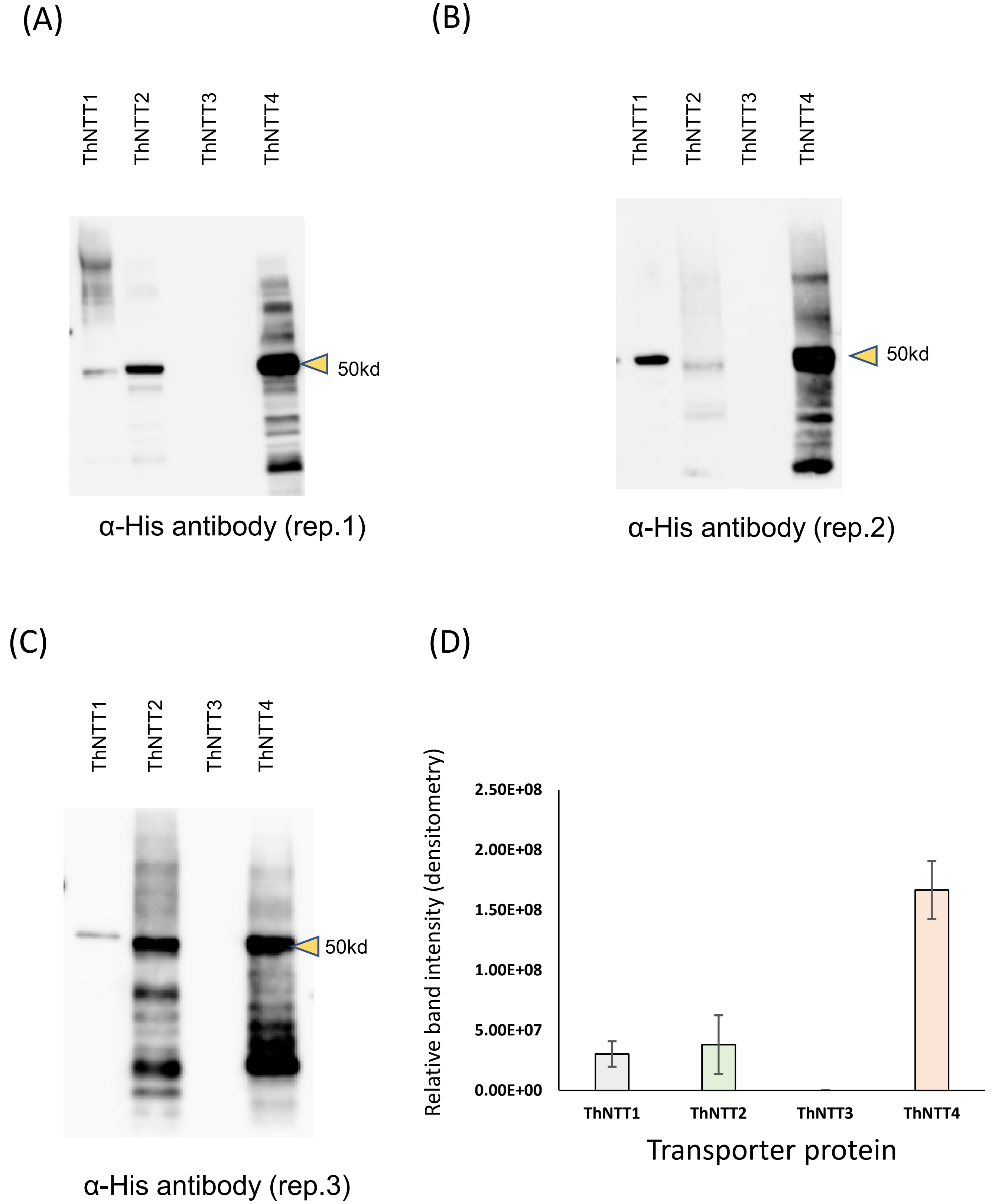

Supplementary figure 1. Western blot analysis of NTT expression in *E. coli* BL21 pLysS. Gene expression was induced and bacteria lysed as described in the Methods section. Data shows 3 representative Western blots (A-C) and densitometry data (D) of the Western signal using Biorad densitometry software. The transporter protein ThNTT4 exhibited consistent and high levels of expression in all replicates, with variable and lower levels of expression for ThNTT1-3, as supported by the densitometry data (D). Data shows mean ± SE from 3 biological replicates.

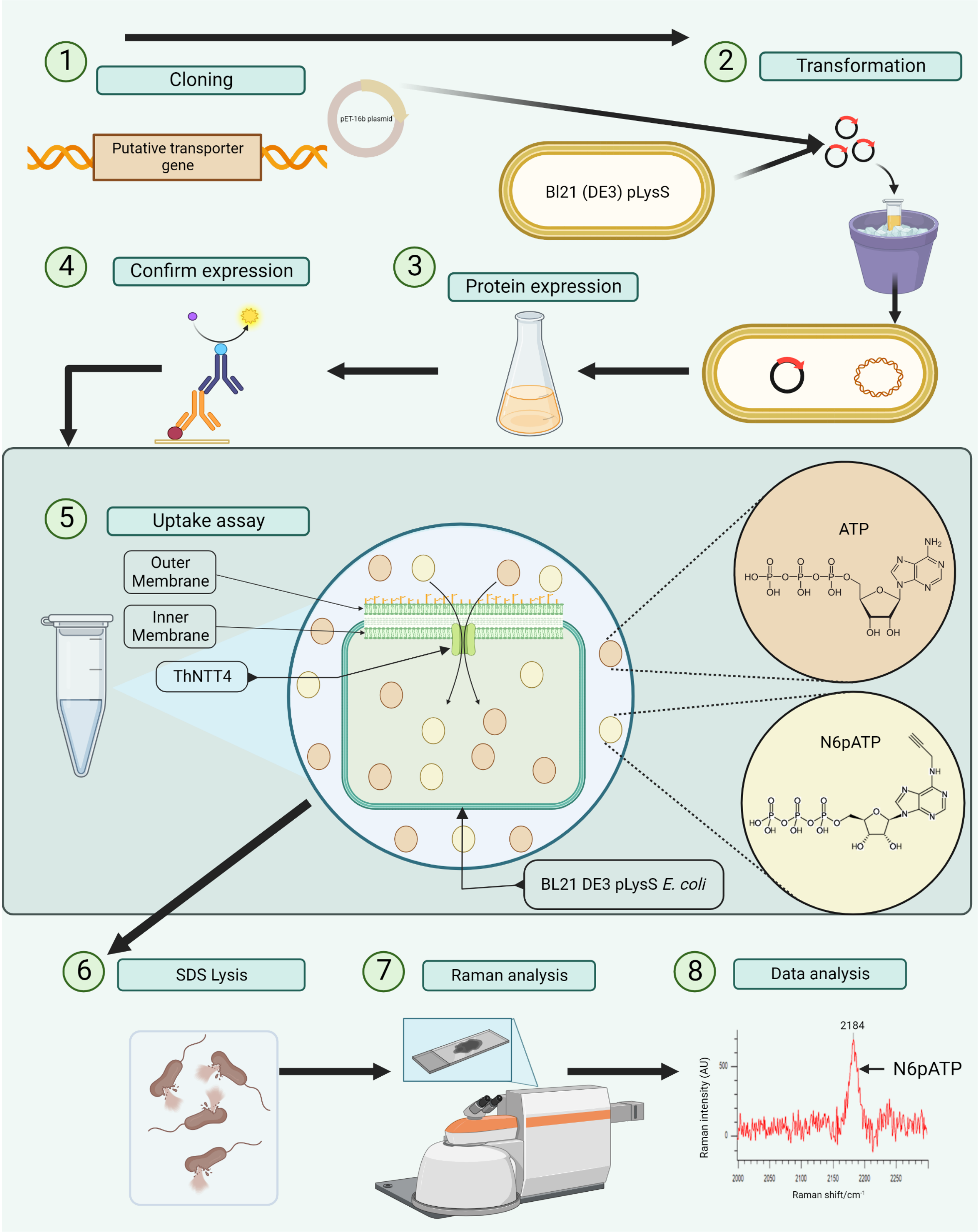

Supplementary Figure 2. Schematic showing the overall procedure for uptake assay of alkyne labelled substrates using Raman microscopy. A putative transporter gene (ThNTT4) is cloned into a suitable expression vector (1) and transformed into a heterologous expression system (2). Protein expression is induced (3) and confirmed (4), followed by an uptake assay using alkyne-labelled substrates such as N6pATP (5). Following washing and lysis of the heterologous host cell (6), the lysate is spotted onto a steel slide, dried down and analysed with the Raman microscope. Detailed methodology is given in the paper.
